# Notch Regulates Macrophage-Mediated Inflammation in Diabetic Wound Healing

**DOI:** 10.3389/fimmu.2017.00635

**Published:** 2017-06-01

**Authors:** Andrew S. Kimball, Amrita D. Joshi, Anna E. Boniakowski, Matthew Schaller, Jooho Chung, Ronald Allen, Jennifer Bermick, William F. Carson, Peter K. Henke, Ivan Maillard, Steve L. Kunkel, Katherine A. Gallagher

**Affiliations:** ^1^Department of Surgery, University of Michigan, Ann Arbor, MI, United States; ^2^Department of Pathology, University of Michigan, Ann Arbor, MI, United States; ^3^Department of Medicine, University of Michigan, Ann Arbor, MI, United States; ^4^Department of Pediatrics, University of Michigan, Ann Arbor, MI, United States

**Keywords:** wound healing, macrophages, Notch, diabetes, inflammation

## Abstract

Macrophages are essential immune cells necessary for regulated inflammation during wound healing. Recent studies have identified that Notch plays a role in macrophage-mediated inflammation. Thus, we investigated the role of Notch signaling on wound macrophage phenotype and function during normal and diabetic wound healing. We found that Notch receptor and ligand expression are dynamic in wound macrophages during normal healing. Mice with a myeloid-specific Notch signaling defect (*DNMAML^floxed^Lyz2^Cre+^*) demonstrated delayed early healing (days 1–3) and wound macrophages had decreased inflammatory gene expression. In our physiologic murine model of type 2 diabetes (T2D), Notch receptor expression was significantly increased in wound macrophages on day 6, following the initial inflammatory phase of wound healing, corresponding to increased inflammatory cytokine expression. This increase in *Notch1* and *Notch2* was also observed in human monocytes from patients with T2D. Further, in prediabetic mice with a genetic Notch signaling defect (*DNMAML^floxed^Lyz2^Cre+^* on a high-fat diet), improved wound healing was seen at late time points (days 6–7). These findings suggest that Notch is critical for the early inflammatory phase of wound healing and directs production of macrophage-dependent inflammatory mediators. These results identify that canonical Notch signaling is important in directing macrophage function in wound repair and define a translational target for the treatment of non-healing diabetic wounds.

## Introduction

According to the CDC, over 1 in 10 Americans suffers from type 2 diabetes (T2D) (1). As the diabetes epidemic exponentially grows, secondary complications of diabetes, such as impaired wound healing, are increasing disproportionately. Non-healing T2D wounds represent a significant economic burden, costing 200 billion dollars annually. Impaired wound healing is among the leading causes of hospital admission in T2D patients and is the leading cause of lower extremity amputation (2). Importantly, amputations in these patients lead to drastic increases in morbidity and mortality as the 5-year mortality rate postamputation is over 50% (2–5).

Wound healing is a complex, dynamic process that progresses through distinct phases of hemostasis, inflammation, proliferation, and remodeling. The inflammatory phase of wound healing is largely driven by monocyte-derived macrophages recruited from the bone marrow (3, 6, 7). Macrophages have diverse functions in tissue depending on the local environment and other systemic factors. In wounds, macrophages exist along a spectrum where M1 “classically activated” macrophages contribute to tissue inflammation and bactericidal activity, and M2 “alternatively activated” macrophages promote reduction of inflammation, growth factor secretion, and tissue repair (8–10). During normal wound healing, recruited macrophages initially demonstrate a pro-inflammatory phenotype that clears debris while recruiting additional inflammatory cells. As wound healing progresses, the macrophages present in the wound tissue become predominantly anti-inflammatory to promote tissue repair (5, 11). In diabetic wounds, the pro-inflammatory to anti-inflammatory macrophage phenotypic switch is impaired and contributes to chronic inflammation and delayed wound healing (5, 12, 13). The etiology of this divergent macrophage behavior in diabetes is presently unknown, but is likely multifactorial.

The importance of the Notch signaling pathway has been well established in cell fate and development; however, recent studies have defined a role for Notch signaling in myeloid differentiation and inflammation (14, 15). In humans and mice, there are four Notch receptors (Notch1–4) and 5 ligands (Delta-like 1, 3, 4, and Jagged 1 and 2). Once bound by ligand, Notch receptors undergo proteolytic cleavage events that result in the release of Notch intracellular domain (NICD) which translocates to the nucleus where it functions like a transcription factor and complexes with the DNA binding protein CSL, Mastermind (MAML), and histone acetyltransferases, to promote transcription of Notch target genes (14, 16). Maillard et al. developed a novel dominant-negative Mastermind mutant—Dominant Negative Mastermind Like Protein 1 (DNMAML)—that, when expressed, blocks downstream transcription of Notch target genes (17).

A recent study demonstrated that increased Notch signaling in macrophages contributes to atherosclerotic plaque formation through increased inflammation (18). Further, a study by Outtz et al. found that Notch 1 plays a role in F4/80 resident wound macrophages in normal wound healing (19). Given that Notch has been recently shown to play a role in innate immune-mediated inflammation, we investigated whether Notch signaling influences the phenotype and function of recruited macrophages with respect to inflammation, in normal and diabetic wound healing (18, 20). Using a murine model of wound healing, we found that Notch receptor and ligand expression is dynamic during normal wound healing. We then generated a myeloid-specific mouse model that inhibits Notch-mediated transcriptional activation *in vivo* with a green fluorescent protein (GFP)-tagged dominant-negative Mastermind-like 1 protein (*DNMAML^floxed^Lyz2^Cre+^*). In these mice, we show that Notch signaling in macrophages is critical for the initial inflammatory response in wound healing. Further, in our murine model of T2D [diet-induced obese (DIO)], we found that Notch receptor signaling is altered in wound macrophages resulting in increased late wound inflammation and impaired healing. Hence, genetic blockade of Notch signaling in macrophages in our diabetic murine model results in improved late wound repair. Taken together, these results confirm a role for Notch signaling in wound macrophage-mediated inflammation during normal and diabetic wound healing. Importantly, our data suggests that the pathologic Notch signaling seen in diabetic wound macrophages at late time points could be a potential therapeutic target to improve healing.

## Materials and Methods

### Mice

All mice were maintained in the pathogen-free animal facility at the University of Michigan and all experiments were conducted under approval of the Institutional Animal Care and Use Committee. C57BL/6 mice (Jackson Laboratory, Bar Harbor, ME, USA) were purchased and maintained in breeding pairs by the University of Michigan Unit for Laboratory and Animal Medicine. Male offspring were maintained on high-fat diet (HFD) (60% kcal fat, Research Diets) chow or normal diet (ND) (13.5% kcal fat; lab diet) chow for 12–16 weeks to induce the DIO model of metabolic syndrome and pre-diabetes in HFD-fed mice. To inhibit Notch signaling in myeloid cells, we crossed *DNMAML^floxed^* mice on a C57BL/6 background (courtesy of Ivan Maillard, MD; University of Michigan) with *Lyz-2^Cre^* mice purchased from Jackson Laboratories (17). Expression of Cre recombinase results in *DNMAML-GFP* expression from the ubiquitously active *ROSA26* promoter. Animals were backcrossed for five generations. Genetoyping was performed by both PCR and flow cytometry for GFP. Animals underwent all experiments between 22 and 26 weeks of age.

### Wound Healing Model

Mice were anesthetized IP with a ketamine/xylazine mixture, dorsal fur was removed with Veet™ Hair Removal Cream and skin was cleaned with sterile water. Full-thickness 4 mm punch biopsy wounds were created on two locations on the mid-back approximately 1 cm apart on the dorsal skin as previously described (5). Wounds were harvested at various time points with a 6 mm punch biopsy for histologic, qPCR, and flow cytometry analysis.

### Assessment of Wound Healing

All wound healing assessments were performed by two to three blinded reviewers and independent verification was performed as previously described (5). Mice were anesthetized and wound surface area was recorded daily using an 8mp digital iPad camera. Internal scales were used in the photographs and wound area was calculated using NIH ImageJ Software (National Institute of Heath, Bethesda, MD, USA). Initial wound size was calculated immediately after wounding, and wound closure was assessed over time as the percentage of initial wound area.

### Histology

Whole wounds were excised from the midbacks of the mice using a 6 mm punch biopsy on post-injury day 4. Wound sections were fixed in 10% formalin overnight before embedding in paraffin. 5 µM sections were stained with hematoxylin and eosin for evaluation of reepithelialization and granulation. Sections were also stained with Mason’s Trichrome for evaluation of eosin for evaluation of reepithelialization and granulation. Sections were also stained with Mason’s Trichrome for evaluation of collagen deposition. Images were captured using Olympus BX43 microscope and Olympus CellSens Dimension software. Percent reepithelialization was calculated by measuring distance traveled by epithelial tongues on both sides of wound divided by total distance needed for full reepithelialization.

### Cell Culture

For all *in vitro* studies, mouse femurs and tibias were flushed with cold RPMI. Cells were then counted and plated in RPMI, FBS, L-Cell Supernatant, Glutamine, P/S to yield a 99.9% homogenous plate of bone marrow-derived macrophages (BMDM) as confirmed by flow cytometry and described previously (8, 11, 21). BMDMs were replated at day 7, 24 h prior to stimulation with media, lipopolyssacharide (LPS) (100 ng/mL), and gamma-secretase inhibitor (GSI) (1 ng/mL) or DMSO. Cells were collected 6 h after stimulation for *in vitro* flow cytometry or treated with Trizol™ (ThermoFisher Scientific) for RNA isolation.

### RNA Extraction

Total RNA extraction was performed with Trizol™ using the manufacturer’s instructions. Briefly, total RNA was reverse transcribed to cDNA using either iScript™ (BioRad) or Superscript III™ Reverse Transcriptase (Thermo Fisher Scientific). qPCR was performed with 2× Taqman PCR mix using the 7500 Real-Time PCR system. Primers for IL1β, IL12, TNFα, Hes1, Hey1, and nitric oxide synthase 2 (NOS2) were purchased from Applied Biosciences. Fold expression was calculated by normalizing to control 18S ribosomal RNA using (2^ΔCt^) comparative threshold analysis. All standards and samples were assayed in triplicate. Data were compiled in Microsoft Excel (Microsoft) and presented using Prism software (GraphPad).

### *In Vivo* Wound Cell Isolation

Wounds were collected with a 6 mm punch biopsy, minced and digested in Collagenase A (Liberase™, Sigma Aldrich) and DNAse I (Sigma Aldrich) at 37°C for 30 min. Wound cell suspensions were gently plunged with a syringe and filtered through a 100 µm filter to yield a single-cell suspension. Single-cell suspensions were then directly stained for flow cytometry or passed through a magnetic-activated cell (MAC) sorting column. For the MACs sorting, single-cell suspensions were incubated with anti-CD19, anti-CD3, and anti-Ly6G magnetic beads and then passed through a MAC sorting column for negative selection (Miltenyi Biotec). The eluent was then collected and incubated with anti-CD11b magnetic beads and passed through a second column to capture the CD11b^+^ cells for positive selection. The columns were then removed from the magnets to elute the CD11b^+^ (CD3^−^, CD19^−^, Ly6G^−^) cells for further analysis.

### *Ex Vivo* Cell Culture for Flow Cytometry

For *ex vivo* intracellular flow cytometry, single-cell wound suspensions were plated in single-housed Teflon-coated wells with complete media (RPMI, 10% FBS, glutamine, and Pen/Strep) for 60 min. GolgiStop™ was then added to stop golgi-mediated protein transport (BD Biosciences; Cat. 51-2092KZ; 1:2,000 dilution). After 120 min with GolgiStop™, cells were then washed and processed for flow cytometry.

### Flow Cytometry

Wound cells were first stained with a LIVE/DEAD^®^ Fixable Yellow Dead Cell Stain Kit (Molecular Probes by Life Technologies; Ref. L34959; 1:1,000 dilution) and then washed two times with cold PBS. Cells were then resuspended in Flow Buffer (500 mL PBS, 0.5 g BSA, 5 mL 2% NaN3 in water, and 5 mL of 1 M Hepes Buffer) and Fc-Receptors were blocked with anti-CD16/32 (BioXCell, Cat. CUS-HB-197, 1:200 dilution) prior to surface staining. Monoclonal antibodies used for surface staining included: Anti-CD3e-Biotin (Biolegend, Cat. 100304, 1:400 dilution), Anti-CD19-Biotin (Biolegend, Cat. 115504, 1:400 dilution), Anti-Ter-119-Biotin (Biolegend, Cat. 116204, 1:400 dilution), Anti-NK1.1-Biotin Cat. 108704, 1:400 dilution), Anti-Ly6G-Biotin (Biolegend, Cat. 127604, 1:400 dilution), Anti-CD11b-PerCP (Biolegend, Cat. 101230, 1:400 dilution), Anti-Ly6C-BV605 (Biolegend, Cat. 128035, 1:400 dilution), Anti-Notch1-BV421 (Biolegend, Cat. 130615, 1:200 dilution), Anti-Notch2-PE (Biolegend, Cat. 130707, 1:200 dilution), and Anti-DLL4-PE (Biolegend, Cat. 130808, 1:200 dilution). Following surface staining, cells were washed twice, and biotinylated antibodies were labeled with streptavidin-APC-Cy7 (Biolegend, Cat. 405208, 1:1,000 dilution). Next, cells were either washed and acquired for surface-only flow cytometry, or were fixed with 2% formaldehyde and then washed/permeablized with BD perm/wash buffer (BD Biosciences, Ref. 00-8333-56) for intracellular flow cytometry. After permeablization, intracellular stains included: anti-IL1β-Pro-PE Cy7 (eBioscience, Ref. 25-7114-82, 1:200 dilution) and anti-TNFα-APC (Biolegend, Cat. 506308, 1:200 dilution). After washing, samples were then acquired on a 3-Laser Novocyte Flow Cytometer (Acea Biosciences). Data were analyzed using FlowJo software version 10.0 (Treestar) and compiled using Prism software (GraphPad). To verify gating and purity, all populations were routinely back-gated.

### Isolation of Human Monocytes from Peripheral Blood

Peripheral blood was obtained from patients with T2D and non-diabetic controls under University of Michigan institutional review board-approved protocols (HUM# 00060733). There were no statistical differences between the groups with respect to demographics or comorbid conditions. Immune cell isolation was performed immediately after the blood was obtained. Blood underwent red blood cell lysis followed by Ficoll separation. Peripheral blood mononuclear cells were harvested from the buffy coat and monocytes were isolated by performing MAC sorting with anti-CD14 magnetic beads. Monocytes were then placed in Trizol™ for qPCR analysis.

### Statistical Analysis

Differences between multiple groups were evaluated using a one-way analysis of variance followed by Newman–Keuls *post hoc* test. All other comparisons were done with a Mann–Whitney *U*-test and a two-tailed Student’s *t*-test. Data are expressed as mean ± SEM. All data are representative of at least three independent experiments. A *P*-value of 0.05 or less was considered statistically significant.

## Results

### Notch Receptor Expression Levels Are Dynamic during Wound Healing

**Figure 1:**
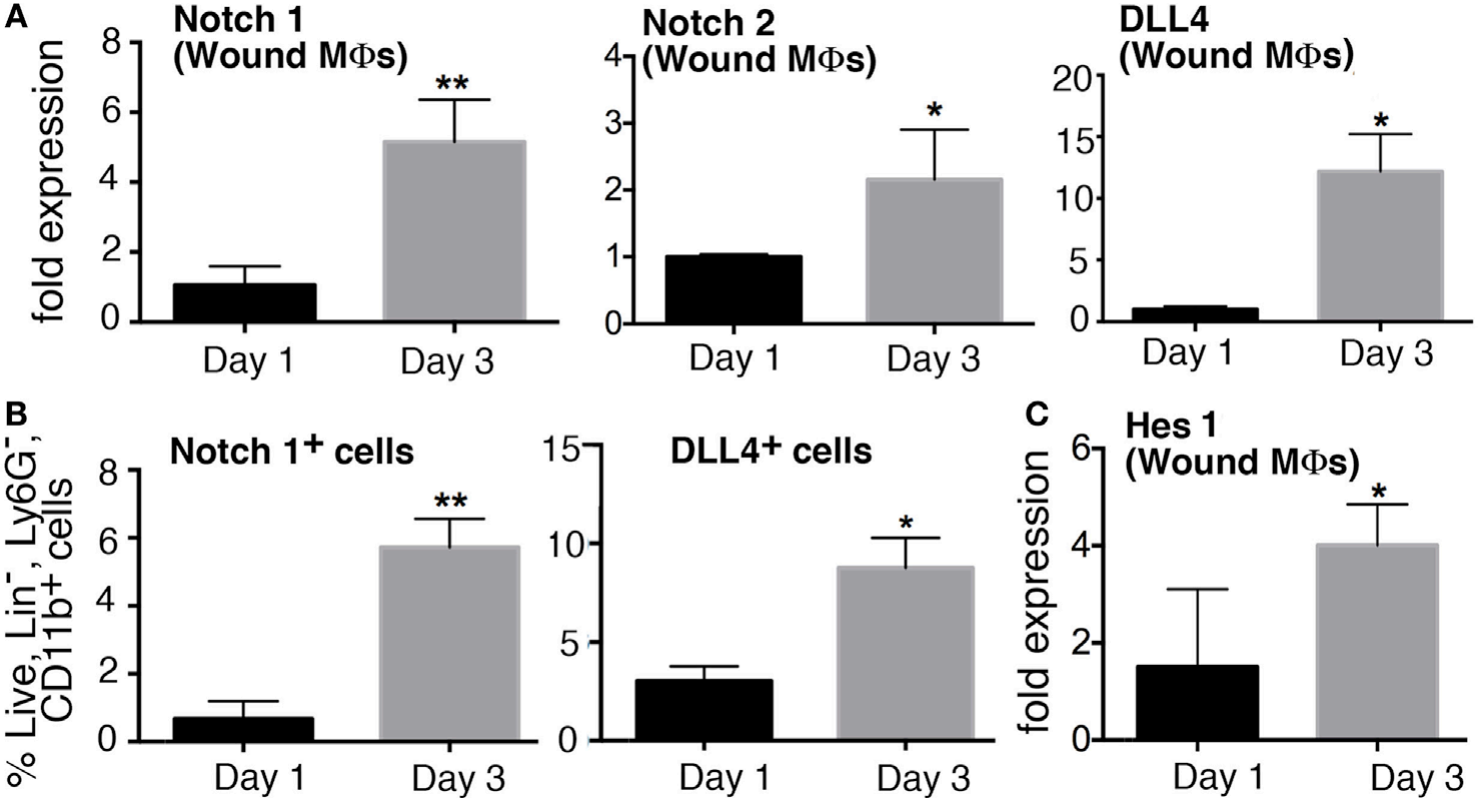
**Notch signaling is dynamic in macrophages during early wound healing**. C57Bl/6 mice were wounded on the back using a 4 mm punch biopsy. Wounds were collected on days 1 and 3 post-wounding. **(A)** Wound macrophages (CD3^−^/CD19^−^/Ly6G^−^/CD11b^+^) were isolated using magnetic-activated cell (MAC) sorting on days 1 and 3 post-wounding. *Notch 1* receptor, *Notch 2* receptor, and *DLL4* ligand expression quantified by RT-PCR on days 1 and 3 (*n* = 10 wounds in 5 mice/group; replicated 3×) (*P < 0.05; **P < 0.01). **(B)** Flow cytometry quantification of Notch receptors/ligands in wounds on days 1 and 3. Notch 2 and DLL4 positive cells on days 1 and 3 expressed as a percent of live, lin^−^,Ly6G^−^,CD11b^+^ cells (*n* = 12 wounds in 6 mice/group; replicated 2×) (*P < 0.05; **P < 0.01). **(C)** *Hes1* gene expression was quantified by RT-PCR on days 1 and 3 (*n* = 10 wounds in 5 mice/group; replicated 3×) (*P < 0.05). Statistical analysis was performed using two-tailed Student’s *t*-test. All data are expressed as mean ± SEM.

Since inflammation is critical in the early stages post-injury to direct the healing cascade and Notch signaling plays a role in inflammation in atherosclerosis and other diseases, we sought to determine if Notch receptors/ligands were dynamic in macrophages during early wound healing (18, 19). Wounds were created in C57BL/6 mice and harvested on days 1, and 3 post-wounding and CD11b^+^ (Ly6G^−^, CD19^−^, CD3^−^) macrophages were isolated by MACs sorting. Expression of receptors (*Notch1–4*) and ligands (*DLL1, 4* and *Jagged1*) were examined in wound macrophages. *Notch1, Notch2*, and *DLL4* expression was significantly increased in wound macrophages on day 3 compared to day 1 (Figure 1A). Notch1, Notch2, and DLL4 ligand have all been previously found to play a role in macrophage-mediated inflammation; hence, this alteration in receptor/ligands during the inflammatory phase of wound healing was not unexpected. Since we found that *Notch1, Notch2*, and *DLL4* expression was dynamic during wound healing in macrophages over time, we performed flow cytometry to determine if these gene expression findings corresponded to receptor/ligand protein in macrophages. Flow cytometry was performed on wounds at days 1 and 3 post-injury. NOTCH1 and DLL4 were found to significantly increase from day 1 to 3 in normal wound tissue (Figure 1B). In order to correlate these findings with downstream Notch signaling, we examined *Hes1* expression and found it was increased at day 3 post-wounding (Figure 1C). These data suggest that Notch signaling is dynamic in macrophages during wound healing and may play a role in macrophage function during the early inflammatory phase.

### *DNMAML^floxed^Lyz2^Cre+^* Genotype Results in a Myeloid-Specific Blockade of Downstream Canonical Notch Signaling in Peripheral Wounds

**Figure 2:**
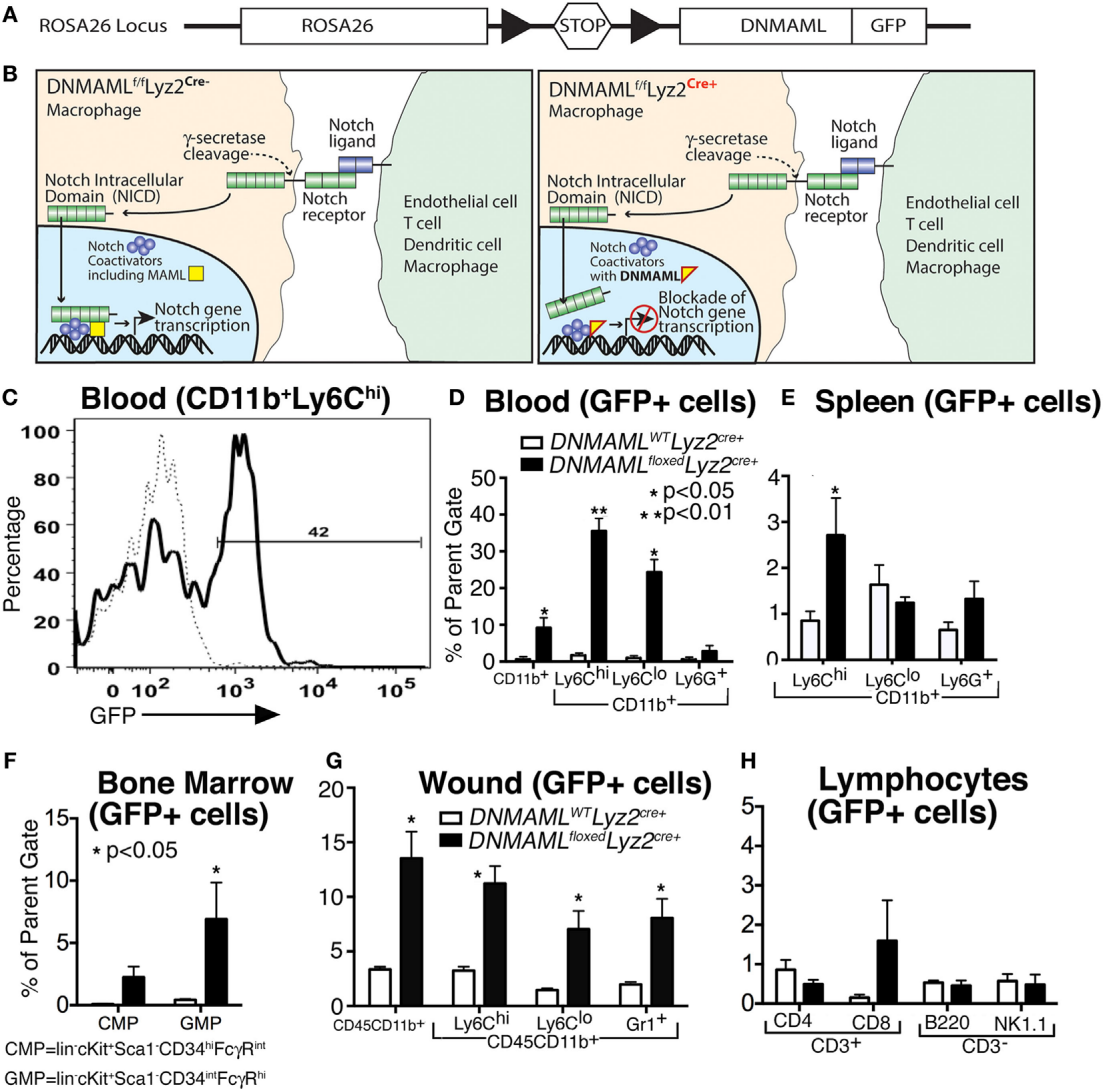
**Generation of a macrophage-specific, Notch signaling-deficient murine model (*DNMAML^floxed^Lyz2^Cre+^*) with myeloid specificity in tissues**. **(A)** Gene construct representing *DNMAML^floxed^* mice on a C57Bl/6 background, where crossing mice with C57Bl/6-Lyz2^Cre+^ will result in excision of a stop codon and transcription of DNMAML-GFP from the Rosa26 Locus in macrophages. **(B)** Schematic demonstrating blockade of downstream Notch signaling following receptor/ligand interaction in *DNMAML^floxed^Lyz2^Cre+^* mice. **(C)** Representative example of flow cytometry for green fluorescent protein (GFP) in CD11b^+^ Ly6C^hi^ cells isolated from peripheral blood in the *DNMAML^floxed^Lyz2^Cre+^* mice. **(D–G)** Percentage of GFP^+^ cells in myeloid cell populations examined by flow cytometry from *DNMAML^floxed^Lyz2^Cre+^* mice and littermate controls in **(D)** peripheral blood, **(E)** spleen, **(F)** bone marrow, and **(G)** peripheral wounds (*n* = 5 mice/group, replicated 2×) (*P < 0.05;**P < 0.01). **(H)** Percentage of GFP^+^ cells in lymphocyte populations from spleen examined by flow cytometry from *DNMAML^floxed^Lyz2^Cre+^* mice and littermate controls (*n* = 5 mice/group, replicated 2×). All data are expressed as mean ± SEM.

In order to explore the role of Notch signaling in macrophages in wound healing, we obtained a mouse with a cassette encoding the Notch inhibitor, Dominant Negative Mastermind Like Protein 1 (DNMAML) fused with a GFP at the *ROSA26* locus downstream of a floxed stop codon sequence that prevents transcription of DNMAML (Figure 2A) (17). We then crossed these mice with Lyz2-Cre mice on a C57BL/6 background to create a mouse strain with myeloid-specific expression of DNMAML-GFP in the Cre^+^ mice. Expression of the DNMAML protein blocks NICD from binding to transcription factors and prevents downstream transcription of Notch target genes (Figure 2B).

In order to demonstrate the cell specificity of these newly generated *DNMAML^floxed^Lyz2^Cre+^* mice, flow cytometry was performed to examine GFP expression in myeloid and non-myeloid cell subsets in bone marrow, peripheral blood, spleen, and wound tissue (Figures 2C–G). Importantly, CD11b^+^ Ly6C^hi^ monocytes in peripheral blood had the highest expression of GFP among the various myeloid cell populations (Figure 2D). Similar trends were seen in CD11b^+^ Ly6C^hi^ cells in spleen, bone marrow and wound tissue (Figures 2E–G). Significantly less GFP was seen in other non-myeloid cells and in control (*DNMAML^WT^Lyz2^Cre+^*) mice confirming the macrophage specificity and functionality of the model for Notch signaling in macrophages (Figures 2D–H). Hence, in our murine model of macrophage-specific blockade of downstream Notch signaling, we are able to assess the role of Notch in macrophages in normal and pathologic wound healing.

### Early Wound Healing Is Delayed in *DNMAML^floxed^Lyz2^Cre+^* Mice and Is Associated with Decreased Inflammatory Wound Macrophages

**Figure 3:**
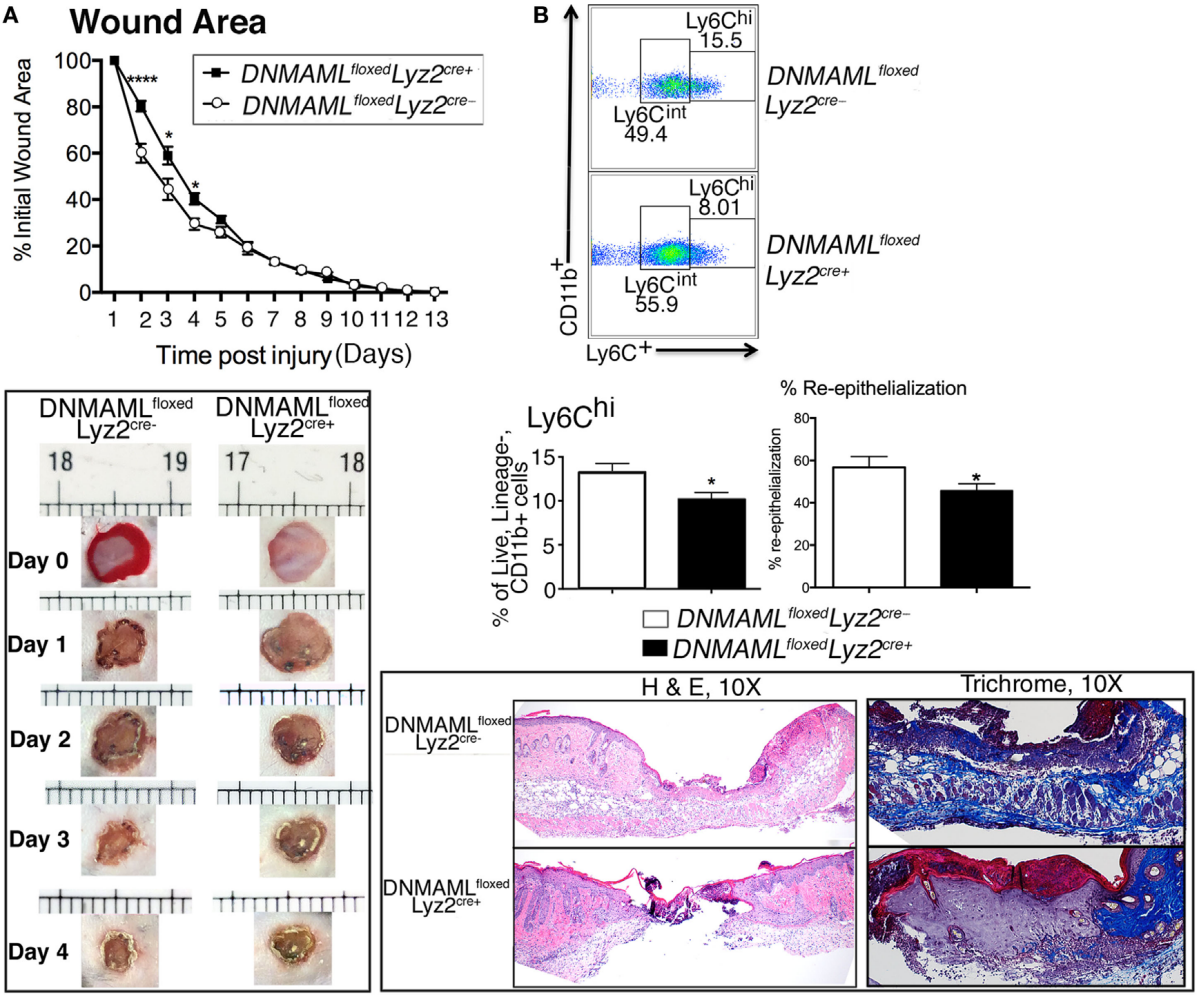
**Genetic blockade of canonical Notch signaling in macrophages results in decreased inflammatory wound macrophages and delayed early wound healing**. Wounds were created using 4 mm punch biopsies on the backs of *DNMAML^floxed^Lyz2^Cre+^*and littermate control mice. **(A)** Change in wound area was recorded daily using ImageJ Software (NIH) until complete healing was observed. Data are pooled from two experiments (*n* = 20 wounds in 10 mice/group, repeated 3×) (*P < 0.05; ****P < 0.0001). Histopathology (H&E) and collagen deposition of wounds of *DNMAML^floxed^Lyz2^Cre+^*and littermate controls on post-injury day 4. *DNMAML^floxed^Lyz2^Cre+^*wounds showed less reepithelialization, granulation formation, and collagen deposition. Data are pooled from two experiments (*n* = 20 wounds in 10 mice/group, repeated 2×). **(B)** Analytical flow cytometry of Lin^−^/Ly6G^−^/CD11b^+^/Ly6C^hi^ wound cells in from *DNMAML^floxed^Lyz2^Cre+^* mice and littermate controls at day 3 post-injury (*n* = 10 wounds in 5 mice/group, repeated 2×) (*P < 0.05). Statistical analysis was performed using two-tailed Student’s *t*-test. All data are expressed as mean ± SEM.

To determine the effect of Notch signaling in macrophage function during wound healing, we first wounded *DNMAML^floxed^Lyz2^Cre+^* and littermate control mice and analyzed their healing rates. Specifically, two full-thickness wounds were created symmetrically on the backs of mice and wounds were analyzed throughout the healing process. We found markedly delayed early wound closure in the *DNMAML^floxed^Lyz2^Cre+^* mice compared to littermate controls with statistically significant differences at early post-injury days 2–4 (Figure 3A). Histological assessment showed that the wounds from *DNMAML^floxed^Lyz2^Cre+^* mice had less reepithelialization on the edge of the wound as well as granulation tissue formation. Further trichrome staining showed less collagen deposition in *DNMAML^floxed^Lyz2^Cre+^* mice compared to littermate controls. These findings confirm the importance of Notch signaling early in the inflammatory phase of wound healing. Based on this finding, we hypothesized that the impaired early wound healing was the result of inadequate early inflammation from infiltrating wound monocyte/macrophages as a consequence of impaired Notch signaling. In order to examine this *in vivo*, we looked at wound cell populations by analytical flow cytometry to determine monocyte/macrophage phenotype at day 3, a critical time point in the initiation of the inflammatory phase of wound healing. Importantly, CD11b^+^ Ly6C^Hi^ cells, were significantly decreased in *DNMAML^floxed^Lyz2^Cre+^* mice compared to littermate controls (Figure 3B). As previously documented, CD11b^+^ Ly6C^Hi^ cells represent infiltrating inflammatory monocyte/macrophages in acute wound healing and are a critical cell population for producing the initial inflammatory response (22–26). Thus, the impaired early healing in our Notch signaling-deficient mice is likely secondary to the decreased number of inflammatory monocyte/macrophages.

### Blockade of Downstream Notch Signaling in Macrophages Decreases Inflammatory Cytokine Production *In Vitro* and *In Vivo*

**Figure 4:**
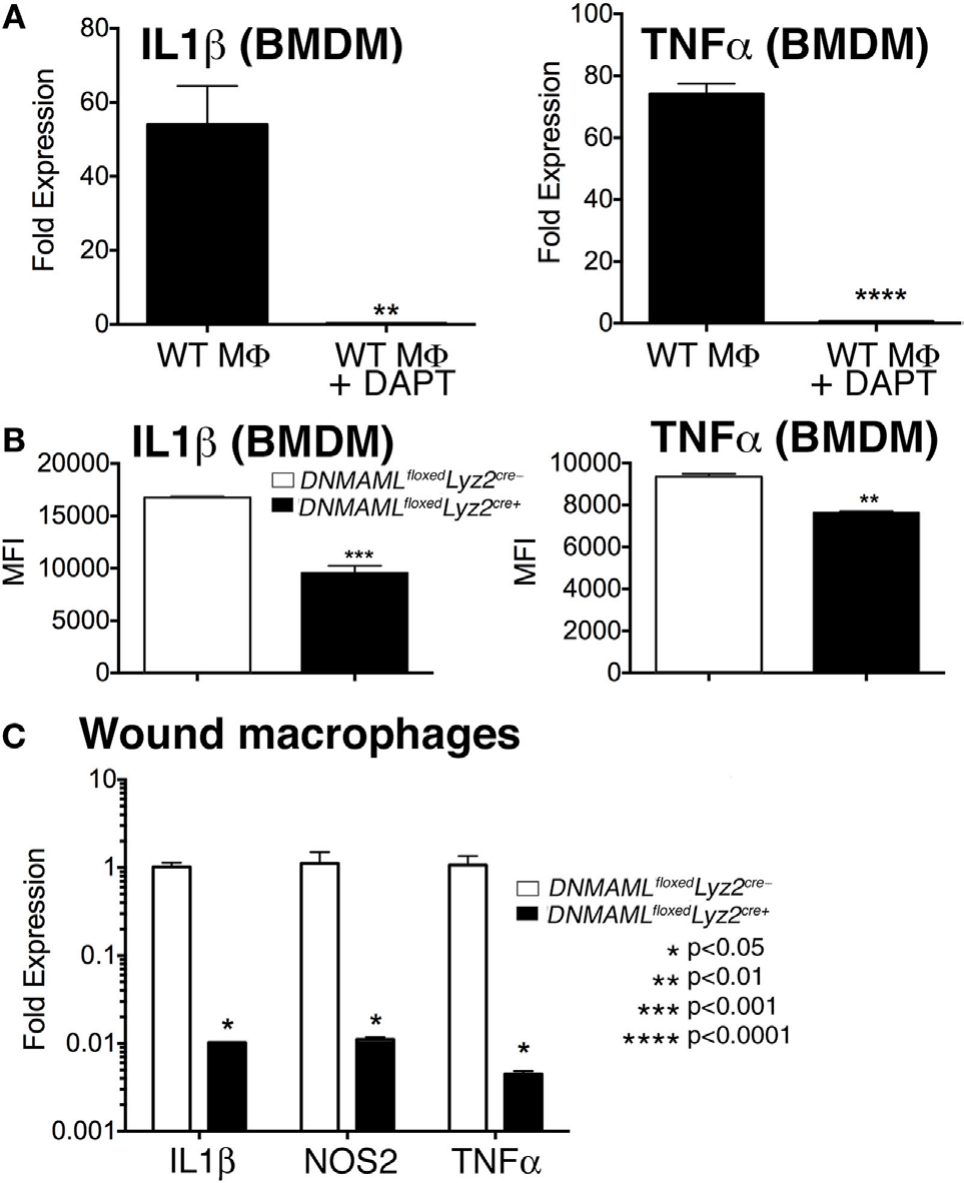
**Genetic and pharmacologic blockade of Notch signaling in macrophages results in decreased inflammatory cytokine production *in vitro* and *in vivo***. **(A)** Bone marrow-derived macrophages (BMDMs) were isolated from C57Bl/6 mice and stimulated with lipopolyssacharide (LPS) (100 ng/mL) for 12 h in the presence or absence of a gamma-secretase inhibitor (*N*-[*N*-(3,5-Difluorophenacetyl)-l-alanyl]-S-phenylglycine t-butyl ester) (DAPT) (1 nM) or DMSO control. *IL1*β and *TNF*α gene expression was quantified by RT-PCR (*n* = 3 mice, repeated in triplicate) (**P < 0.01; ****P < 0.0001). **(B)** BMDMs were isolated from *DNMAML^floxed^Lyz2^Cre+^* and littermate control mice and stimulated with LPS (100 ng/mL) for 12 h. Surface and intracellular stains were performed and cells were then analyzed by flow cytometry for IL1β and TNFα. Results are expressed as mean fluorescence intensity (MFI) (*n* = 3 mice, repeated in triplicate) (**P < 0.01; ***P < 0.001). **(C)** Wounds were created using 4 mm punch biopsies on the backs of *DNMAML^floxed^Lyz2^Cre+^*and littermate control mice. Wound macrophages (CD3^−^/CD19^−^/Ly6G^−^/CD11b^+^) were isolated using magnetic-activated cell (MAC) sorting on day 3 post-wounding. *IL1*β, *TNF*α, and *NOS2* expression in the wound macrophages was quantified by RT-PCR (*n* = 10 wounds in 5 mice/group, repeated 2×) (*P < 0.05). Statistical analysis was performed using analysis of variance and two-tailed Student’s *t*-test. All data are expressed as mean ± SEM.

In order to further define the effect of Notch signaling on macrophage-induced inflammation, we examined expression of inflammatory cytokines *in vitro* and *in vivo* using both genetic and pharmacologic mechanisms of Notch signaling blockade. To examine the effect of pharmacologic Notch blockade on macrophages *in vitro*, we cultured bone marrow-derived macrophages (BMDMs) from C57BL/6 mice. We utilized bone marrow myeloid cells as these cells are known to be taken up in the blood and rapidly recruited to injured tissue where they quickly outnumber tissue-resident macrophages (5, 24, 25, 27–32). BMDMs were then stimulated with LPS, to mimick *in vivo* wound toll-like receptor 4 (TLR4) signaling, and GSI (*N*-[*N*-(3,5-Difluorophenacetyl)-l-alanyl]-S-phenylglycine t-butyl ester [DAPT]) or DMSO control were added to the cells, and inflammatory cytokine expression was analyzed (33–35). Interestingly, those cultured cells that were in the presence of a GSI had 50- to 70-fold less expression of *IL1*β and *TNF*α compared to littermate controls (Figure 4A). In order to examine inflammatory cytokine production in our genetic Notch signaling-deficient macrophages, BMDM from *DNMAML^floxed^Lyz2^Cre+^* and littermate controls were examined by intracellular flow cytometry to look at protein levels. There was a significant decrease in IL1β and TNFα in *DNMAML^floxed^Lyz2^Cre+^* macrophages compared to littermate controls (Figure 4B). These experiments demonstrate that Notch signaling inhibition by both pharmacologic and genetic methods results in decreased inflammatory cytokine production by macrophages.

In order to determine if these *in vitro* decreases in inflammatory mediators correlate with macrophages *in vivo*, we isolated wound CD11b^+^ (Ly6G^−^, CD3^−^, CD19^−^) cells by MAC sorting and analyzed inflammatory cytokine expression. Consistent with what we had demonstrated *in vitro, in vivo* wound macrophages isolated from *DNMAML^floxed^Lyz2^Cre+^* mice made significantly less *IL1*β, *NOS2*, and *TNF*α compared to the littermate controls (Figure 4C). Taken together, these findings suggest that Notch signaling in macrophages is crucial for the generation of an inflammatory response to tissue injury.

### In a Murine Model of Diabetes, Notch Signaling Is Increased in Wound Macrophages During Late Wound Healing

**Figure 5:**
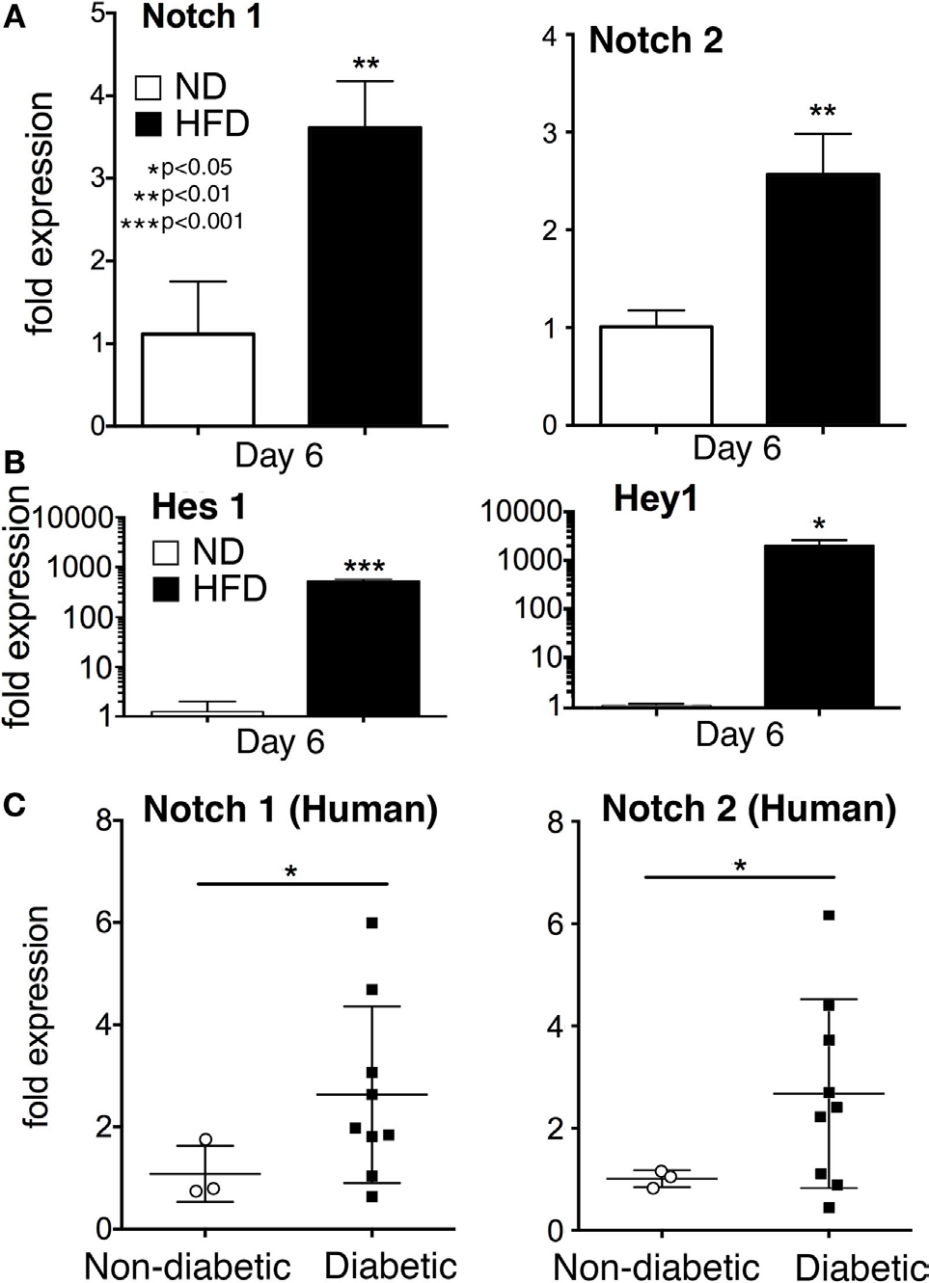
**Notch signaling is increased in monocytes from type 2 diabetic (T2D) patients and wound macrophages from a murine model of obesity/type 2 diabetes (T2D)**. Diet-inducted obesity, a physiologic model of “prediabetes,” was used to examine the effect of obesity and insulin resistance on Notch signaling in wound macrophages. C57Bl/6 mice were fed either normal diet (ND) (12% saturated fat) or a high-fat diet (HFD) (60% saturated fat) for 12–14 weeks. Mice on a HFD (diet-induced obese) were wounded on the back using a 4 mm punch biopsy. Similar to T2D patients, HFD mice have delayed wound healing and thus, wounds were collected on day 6 post-wounding. **(A)** Wound macrophages (CD3^−^/CD19^−^/Ly6G^−^/CD11b^+^) were isolated using magnetic-activated cell (MAC) sorting on day 6 post-wounding. *Notch 1* receptor and *Notch 2* receptor expression was quantified by RT-PCR on day 6 (*n* = 10 wounds in 5 mice/group; replicated 2X) (**P < 0.01). **(B)** Notch target genes, *Hes1* and *Hey1* expression levels was quantified by RT-PCR in wound macrophages (CD3^−^/CD19^−^/Ly6G^−^/CD11b^+^) on day 6 post-injury (*n* = 10 wounds in 5 mice/group, replicated 2×) (*P < 0.05; ***P < 0.001). **(C)** Peripheral blood from patients with T2D and non-diabetic controls was obtained from clinic. Age, gender and comorbid conditions were evenly distributed among the two groups. Following RBC lysis and Ficoll separation, CD14^+^ monocytes were isolated from the buffy coat. *Notch 1* and *Notch 2* receptor expression was quantified by RT-PCR (*n* = 12 patients, replicated 2×) (*P < 0.05). Statistical analysis was performed using two-tailed Student’s t-test. All data are expressed as mean ± SEM.

Impaired healing in T2D is well documented and is due, in part, to a prolonged hyperinflammatory response generated by macrophages. The role of Notch signaling in this process is unknown. To define the effect of Notch signaling in pathologic wound healing characterized by chronic inflammation we utilized the (DIO or HFD) model, as a physiologic model of obesity/T2D. We have previously published that macrophages isolated from HFD wounds produce increased inflammatory cytokines and impair wound healing (5). In order to examine the role of Notch in this hyperinflammatory state, we isolated wound macrophages *via* MAC sorting from HFD mice harvested at a late time point (day 6 post-injury). These HFD wound macrophages demonstrated significantly increased *Notch1* and *Notch2* expression compared to controls (Figure 5A). When we examined the downstream Notch target genes *Hes1* and *Hey1* in these *in vivo* macrophages on day 6, there was a 1,000-fold increase in expression in the HFD wound macrophages compared to controls (Figure 5B). Since loss of Notch signaling decreased inflammatory cytokine expression during early wound healing and presence of Notch signaling was necessary for normal early wound healing, this increased Notch signaling in late wound macrophages in the setting of T2D may partially be responsible for the extended hyperinflammatory response seen in diabetic wounds. In order to translate these findings to human disease, we isolated peripheral blood monocytes (CD14^+^) from patients with and without T2D. Since monocytes are recruited to the wound during repair, we examined human monocytes for Notch receptor expression. We found that in patients with T2D, *Notch1* and *Notch2* were significantly elevated compared to non-T2D control patients (Figure 5C). There were no differences in DLL4 expression between the groups (data not shown). These findings suggest that Notch signaling may play a role in the dysregulated inflammation seen in T2D wounds.

### *DNMAML^floxed^Lyz2^Cre+^* Mice on a HFD Develop Glucose Intolerance and Yield Macrophages That Produce Decreased Inflammatory Cytokines

**Figure 6:**
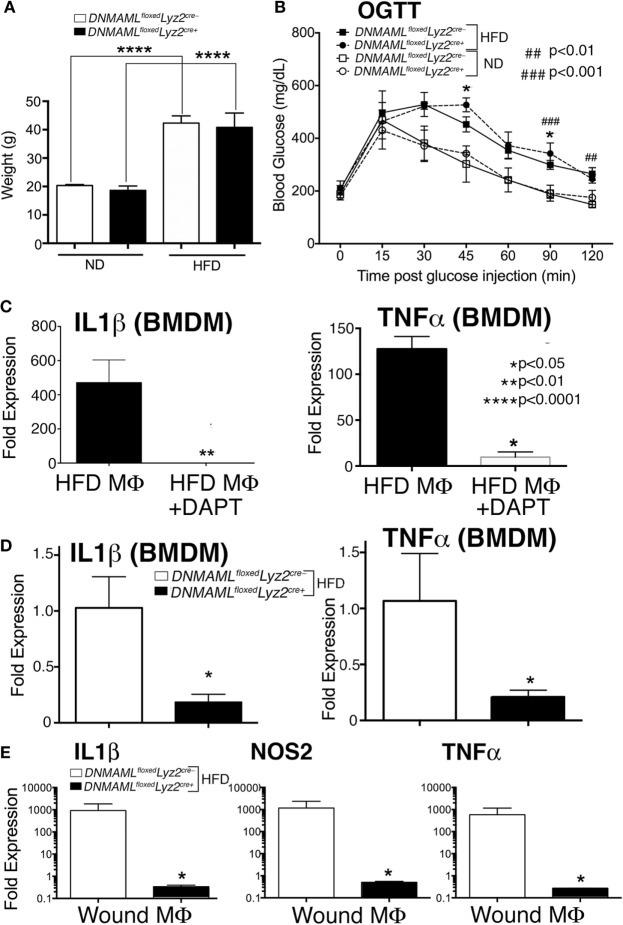
**Notch signaling-deficient mice on a high-fat diet (HFD) (HFD *DNMAML^floxed^Lyz2^Cre+^*) develop obesity and insulin resistance and *in vitro* and *in vivo* macrophages demonstrate decreased inflammation**. *DNMAML^floxed^Lyz2^Cre+^*and littermate control mice were fed either normal diet (ND) (12% saturated fat) or a HFD (60% saturated fat) for 12–14 weeks. **(A)** Weight (grams) of *DNMAML^floxed^Lyz2^Cre+^*and littermate control mice were obtained following 14 weeks of HFD (*n* = 5 mice/group, repeated 2×) (****P < 0.0001). **(B)** Oral glucose tolerance test (OGTT) was performed in all groups. Blood glucose (milligrams/deciliter) was obtained every 15–30 min for 2 h (*n* = 5 mice/group) (*P < 0.05 for littermate ND versus HFD mice; ###*P* < 0.001, ##*P* < 0.01 for *DNMAML^floxed^Lyz2^Cre+^*ND versus HFD mice). **(C)** Bone marrow-derived macrophages (BMDMs) were isolated from C57Bl/6 mice on HFD and stimulated with lipopolyssacharide (LPS) (100 ng/mL) for 12 h in the presence or absence of a gamma-secretase inhibitor (*N*-[*N*-(3,5-Difluorophenacetyl)-l-alanyl]-S-phenylglycine t-butyl ester) (DAPT) (1 nM) or DMSO control. *IL1*β and *TNF*α gene expression was quantified by RT-PCR (*n* = 3 mice, repeated in triplicate) (*P < 0.05). **(D)** BMDMs were isolated from from *DNMAML^floxed^Lyz2^Cre+^*and littermate control mice and stimulated with LPS (100 ng/mL) for 12 h and *IL1*β and *TNF*α gene expression was quantified by RT-PCR (*n* = 3 mice, repeated in triplicate) (*P < 0.05). **(E)** Wounds were created using 4 mm punch biopsies on the backs of HFD *DNMAML^floxed^Lyz2^Cre+^*and littermate HFD mice. Wound macrophages (CD3^−^/CD19^−^/Ly6G^−^/CD11b^+^) were isolated using magnetic-activated cell (MAC) sorting on day 6 post-wounding. *IL1*β, *TNF*α and *NOS2* expression in the wound macrophages was quantified by RT-PCR (*n* = 10 wounds in 5 mice/group, repeated 2×) (*P < 0.05). Statistical analysis was performed using analysis of variance or two-tailed Student’s *t*-test. All data are expressed as mean ± SEM.

Since loss of Notch signaling decreased inflammation and Notch signaling is increased in macrophages from diabetic wounds and human T2D monocytes, we theorized that decreasing Notch signaling in DIO mice, could improve wound repair at later time points when pathologic inflammation impairs healing. *DNMAML^floxed^Lyz2^Cre+^* mice and littermate controls were placed on a HFD for 12–16 weeks and mice were weighed. Both *DNMAML^floxed^Lyz2^Cre+^* and controls had significant weight gain with no differences between the HFD groups (~40 g) compared to the ND group (~20 g) (Figure 6A). To test their insulin resistance, an oral glucose tolerance test was performed and demonstrated a significantly delayed return-to-baseline of blood glucose in the HFD *DNMAML^floxed^Lyz2^Cre+^* mice, similar to the HFD littermate controls, C57BL/6 mice, compared to all ND-fed controls (Figure 6B).

In order to confirm decreased inflammatory cytokine production in the presence of chemical Notch blockade, HFD BMDMs were isolated as described and stimulated with LPS (100 ng/mL), to mimic *in vivo* wound TLR4 signaling. The GSI (*N*-[*N*-(3,5-Difluorophenacetyl)-l-alanyl]-S-phenylglycine t-butyl ester [DAPT]) or DMSO control were then added to the cells, and inflammatory cytokine expression was analyzed. The HFD BMDMs that were in the presence of a GSI had markedly less expression of *IL1*β and *TNF*α compared to littermate controls (Figure 6C). In order to examine inflammatory cytokine production in our genetic Notch signaling-deficient macrophages, BMDM from HFD *DNMAML^floxed^Lyz2^Cre+^* and HFD littermate controls were stimulated with LPS (100 ng/mg) and analyzed for inflammatory cytokine expression. There was a significant decrease in *IL1*β and *TNF*α in *DNMAML^floxed^Lyz2^Cre+^* macrophages compared to littermate controls (Figure 6D).

In order to confirm decreased inflammatory cytokine production in these HFD *DNMAML^floxed^Lyz2^Cre+^* mice compared to HFD controls, we MAC sorted macrophages from wounds of HFD *DNMAML^floxed^Lyz2^Cre+^* mice and HFD controls. As expected, HFD *DNMAML^floxed^Lyz2^Cre+^* mice had significantly decreased levels of *IL1*β, *NOS2*, and *TNF*α compared to the HFD littermate controls (Figure 6E). Therefore, our genetic mouse model can be used to examine the effects of loss of Notch signaling on wound healing in a murine model of diabetes.

### *HFD DNMAML^floxed^Lyz2^Cre+^* Mice Demonstrate Improved Late Wound Healing

**Figure 7:**
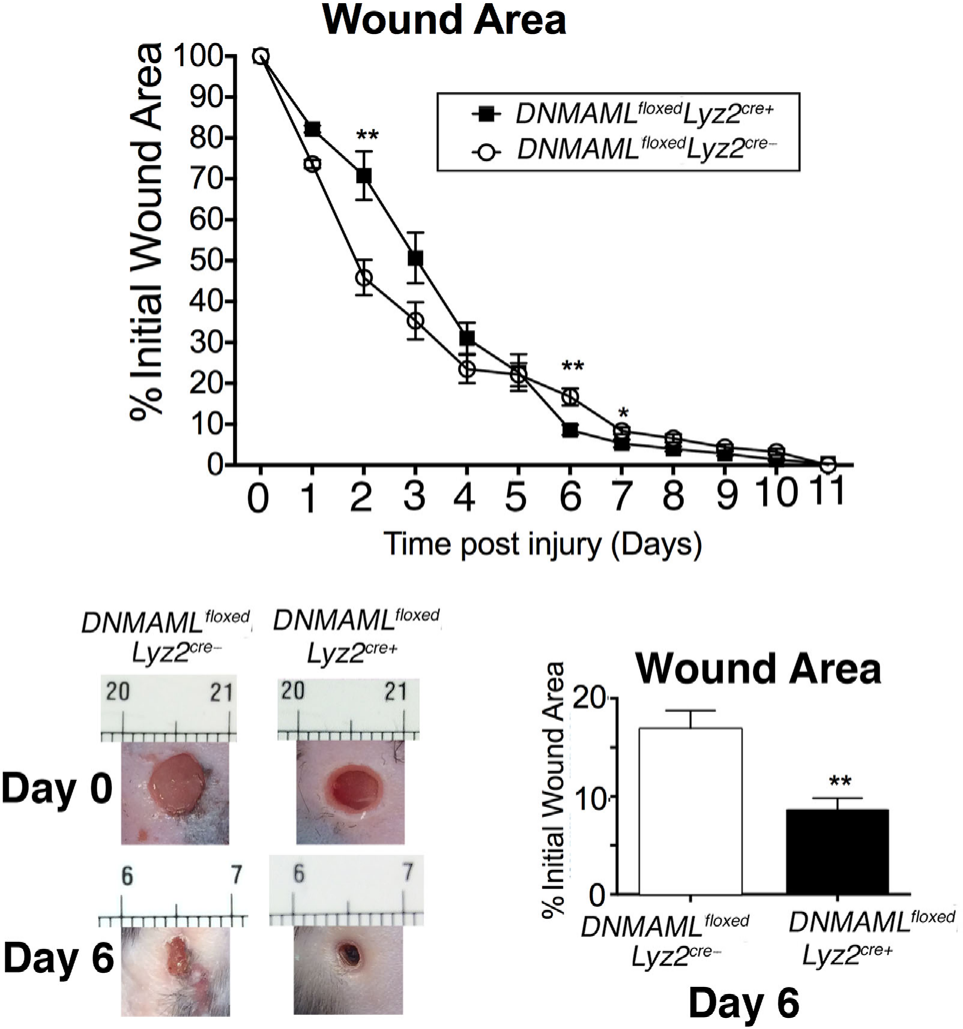
**High-fat diet (HFD) mice deficient in Notch signaling (HFD *DNMAML^floxed^Lyz2^Cre+^)* demonstrate improved late healing compared to HFD controls**. *DNMAML^floxed^Lyz2^Cre+^* and littermate control mice were fed a HFD (60% fat) for 12–14 weeks. Two wounds per mouse were created using 4 mm punch biopsies on the backs of HFD *DNMAML^floxed^Lyz2^Cre+^* and HFD littermate control mice. Change in wound area was recorded daily using ImageJ Software (NIH) until complete healing was observed. Representative images of wounds from days 0 and 6 are shown (*n* = 20 wounds in 10 mice/group) (*P < 0.05; **P < 0.01). Statistical analysis was performed using two-tailed Student’s *t*-test. All data are expressed as mean ± SEM.

As is well established, mice on HFD display impaired wound healing compared to ND controls, which clinically correlates with human wounds in obese T2D patients (5, 36, 37). In order to determine if reduced Notch signaling could improve late wound healing in HFD mice, we examined wound healing in *DNMAML^floxed^Lyz2^Cre+^* mice exposed to HFD for 12–14 weeks. Since early inflammation is essential for normal wound healing progression, we hypothesized that similar to what was observed in the ND *DNMAML^floxed^Lyz2^Cre+^* wound curve (Figure 3B), early healing would be impaired in the HFD *DNMAML^floxed^Lyz2^Cre+^* mice since these mice have decreased inflammatory cytokine expression in their macrophages at all time points. As expected, HFD *DNMAML^floxed^Lyz2^Cre+^* mice had delayed wound healing in the early post-injury period. This difference was less evident in the HFD *DNMAML^floxed^Lyz2^Cre+^* mice since only day 2 had a statistically significant difference while days 3 and 4 trended toward impaired healing in this group. Importantly, there was significantly improved healing in the HFD *DNMAML^floxed^Lyz2^Cre+^* mice at late time points around days 6 and 7 post-injury (Figure 7). Taken together, these findings suggest that increased Notch signaling, and hence, inflammation at late time points in the HFD wounds may be partially abrogated by blockade of canonical Notch signaling.

## Discussion

While the role of macrophages in promoting chronic inflammation in T2D wounds is established, the aberrant signaling pathways that contribute to this dysregulated inflammation remain unknown. The coordinated polarization of wound macrophages at specific times following injury determines whether they promote or prevent tissue repair (3, 5, 12). Although it has recently been identified that Notch signaling plays a role in innate immune cell-mediated inflammation (18), the function of Notch signaling on macrophage plasticity in normal and pathologic tissue repair processes is largely unexplored. In these studies, we have demonstrated the importance of myeloid-specific Notch signaling in normal and pathologic wound healing. Importantly, we have identified the functional consequences of loss of Notch signaling on macrophage phenotype *in vivo* in both normal and diabetic wound tissue. We found that Notch receptors and ligands are dynamic in macrophages during normal wound healing and that loss of Notch signaling results in decreased macrophage-mediated inflammation. Since the establishment of a proper inflammatory response is necessary for regulated wound repair, the blockade of Notch signaling in macrophages resulted in impaired wound healing during the early inflammatory phase, secondary to the decreased ability of macrophages to generate inflammatory cytokines. Interestingly, we observed that Notch receptors and ligands are altered in a physiologic murine model of “prediabetes” with increased Notch signaling at late time points, correlating with increased late inflammation in diabetic wounds. Then finally, we found that the blockade of canonical Notch signaling, resulting in decreased macrophage-mediated inflammation, correlated with improved late wound healing in our model of diabetes.

These findings are intriguing because they draw attention to the importance of the kinetics of inflammation during normal tissue repair. Inflammation, although pathologic in late wound healing, is essential in the early phases of wound healing as it is necessary to initiate the healing response through clearance of debris, removal of pathogens and production of inflammatory mediators that recruit additional immune cells to the wound (38). As wound healing progresses, the macrophages switch from a predominantly inflammatory phenotype to a more reparative phenotype characterized by less inflammatory mediators and more regenerative growth factors (5, 38). A strict timeline of titrated, macrophage-mediated inflammation is necessary for normal wound repair. Our data provide genetic evidence that Notch plays a role in regulating wound macrophage phenotype and inflammation *in vivo* and that Notch signaling is altered in diabetic wound macrophages. To our knowledge, this is the first study to demonstrate altered Notch signaling in pathologic wound healing, and a therapeutic benefit of Notch blockade in late wound healing in the setting of diabetes. It is important to remember that Notch signaling is significantly decreased but not completely blocked in our genetic model; thus, with complete Notch blockade we would expect even greater phenotypic changes than were seen with our floxed-cre dependent knockout.

Since the characterization of resident versus recruited macrophages *in vivo* has changed over the past few years, our paper, in contrast to the 2010 wound study by Outtz et al. reflects the more recent identification of Ly6C as a major marker of monocyte-derived tissue macrophages (19). Though circulating monocyte–macrophages and tissue-resident macrophages represent the same cell type, it has been well documented that these populations are very different with regard to their function within the wound bed (39, 40). In our paper, we are mostly interested in the recruited monocyte–macrophages as they rapidly outnumber tissue-resident macrophages following injury and are thought to be more important in the generation of the inflammatory response (5, 24, 25, 27–32). Another important distinction between these two studies is that although both suggest that Notch plays a critical role in wound healing in both cell populations, this work highlights the potential of a strategic therapeutic Notch signaling blockade to be initiated after the initial inflammatory phase of wound healing in the setting of diabetes. More specifically, as shown in our myeloid-specific Notch signaling-deficient murine model, although blockade of Notch signaling is detrimental to wound healing in the early inflammatory phase, it appears to be protective in late wound healing in a model of diabetes by impeding a prolonged hyperinflammatory response.

Although our study is the first to show that Notch plays a role in recruited monocyte/macrophages in normal and pathologic wound healing, there are several important limitations that should be addressed. While there are multiple factors that influence wound healing, our study focuses on macrophage-mediated inflammation in wounds, thus other processes could have influenced healing in our model. For instance, it is well established by Ansell et al. that the phases of hair follicles surrounding wounds can directly affect wound healing. Specifically, hair follicles in the anagen phase are known to accelerate wound healing by contributing to reepithelialization when compared to telogen or catagen phase hair follicles (41, 42). In order to control for this phenomenon, neonatal mice can be used to allow for synchrony of hair follicles to study the different phases, however, this was not possible in our model due to the need to keep mice on HFD for 12–14 weeks to develop glucose intolerance and insulin resistance and mimic human pre-diabetes. Other limitations include the effect of canonical Notch signaling blockade on other non-inflammation related mediators of wound healing. For example, canonical Notch signaling and vascular endothelial growth factor-A (VEGF-A) are known to play a role in angiogenesis in both tumors and injured tissue, while simultaneously VEGF-A is known to be secreted by infiltrating inflammatory monocytes (25, 43, 44). Thus, it is conceivable that blockade of canonical Notch signaling in our murine wound macrophages may also be affecting angiogenesis. Further studies on the effect of Notch signaling in macrophages in relation to the hair cycle and angiogenesis are needed.

The macrophage response to Notch activation is complicated, and hence, further studies are needed to determine which genes are directly regulated by Notch signaling and how this may affect other signaling pathways that have been shown to work in parallel. It is clear that there are redundant pathways that may contribute to the prolonged inflammatory response in diabetic macrophages. For instance, over expression of NICD1 *in vitro* has been shown to increase TNFα, IL6, and iNOS production *via* rapid degradation of IκBα, increased nuclear translocation of NFκB, and stronger binding of NFκB subunits to the promoters of TNFα and iNOS (45). Other examples involve a signaling loop between Notch receptors and pathogen recognition receptors like TLR4 (46, 47). In one study, deficiency of the terminal Notch signaling transcription factor, RBP-J, attenuated TLR-mediated expression of TNFα, IL6, IL12, and iNOS *in vivo*—suggesting an additive effect of the two cascades (48, 49). Indeed, this is supported by other studies in inflammatory diseases (50), as well as in our current study where we show increased inflammation with increased Notch receptor expression in late diabetic wound healing. Taken together, these findings suggest that Notch signaling is intimately tied to redundant pathways and may act to alter macrophage phenotype, dependent upon the ligands that are present, the receptors bound, and the timing of the interplay between parallel cascades. Further studies are warranted to determine how Notch signaling in T2D may affect TLR and NFκB pathways.

In addition to the effects of Notch signaling on macrophages, Notch likely affects multiple other immune cells and structural cells that play a role in healing. Monocytes, for instance, have been shown to convert from a Ly6C^Hi^, CCR2^+^ phenotype to a Ly6C^Lo^, CX3CR1^+^ phenotype *via* a DLL1–Notch2 signaling directed by the vascular endothelium (18). Further, Notch receptor and ligand expression levels are known to alter T-cell function which may also play an important role in wound healing (51–53). Finally, it has also been shown that Notch1 may play a role in extracellular matrix homeostasis during inflammation by affecting levels of MMP9 (54). Therefore, Notch signaling is likely important in fibroblasts and other structural cells associated with wound repair and may affect inflammation by altering leukocyte migration in inflamed tissues.

Further work is necessary to define the specific downstream Notch target genes that are the most critical for the regulation of inflammation in macrophages during wound repair. Using mice with a myeloid-specific loss of Notch signaling, we have shown that Notch plays a key role in normal wound repair by inciting the generation of classical inflammatory cytokines in wound macrophages. With the concurrent elevation of Notch receptor levels and inflammatory mediators in late wound healing in our murine model of pre-diabetes, and the apparent protective effect of downstream Notch signaling inhibition in our Notch signaling deficient model, it is evident that elevated Notch signaling contributes to the chronic inflammation seen in diabetic wound macrophages. Specific blockade of Notch signaling in macrophages at appropriate time points during wound repair may be beneficial in diabetes to abrogate the chronic inflammation that contributes to impaired wound closure.

## Ethics Statement

Animals: All animals in this study were maintained in a pathogen-free animal care facility at the University of Michigan and all experiments were conducted under approval of the Institutional Animal Care and Use Committee (IACUC). Humans: All human samples and data procured in this study were collected with written informed consent in accordance with the Declaration of Helsinki. Human aspects of this study were conducted under a strict protocol with recommendations and approval from the University of Michigan Institutional Review Board (IRB).

## Author Contributions

KG performed the research, analyzed data, and wrote the manuscript. AK performed the research, analyzed the data, and assisted in writing the manuscript. AJ performed the research. AB, MS, JC, RA, JB, WC, PH, IM, and SK reviewed and edited the manuscript. KG is the guarantor of this work and, as such, had full access to all the data in the study and takes responsibility for the integrity of the data and the accuracy of the data analysis.

## Conflict of Interest Statement

The authors declare that the research was conducted in the absence of any commercial or financial relationships that could be construed as a potential conflict of interest.
